# Importance of Gut Microbiota in Patients with Inflammatory Bowel Disease

**DOI:** 10.3390/nu16132092

**Published:** 2024-06-30

**Authors:** Natalia Ekstedt, Dominika Jamioł-Milc, Joanna Pieczyńska

**Affiliations:** 1 Department of Human Nutrition and Metabolomics, Pomeranian Medical University in Szczecin, 71-460 Szczecin, Poland; natalia.ekstedt@gmail.com; 2 Department of Food Science and Dietetics, Wroclaw Medical University, Borowska 211, 50-556 Wrocław, Poland; joanna.pieczynska@umw.edu.pl

**Keywords:** inflammatory bowel diseases, microbiota, probiotic therapy, ulcerative colitis, Crohn’s disease, probiotic strains

## Abstract

Inflammatory bowel diseases (IBDs), such as Crohn’s disease (CD) and ulcerative colitis (UC), are chronic diseases of the digestive system with a multifactorial and not fully understood etiology. There is research suggesting that they may be initiated by genetic, immunological, and lifestyle factors. In turn, all of these factors play an important role in the modulation of intestinal microflora, and a significant proportion of IBD patients struggle with intestinal dysbiosis, which leads to the conclusion that intestinal microflora disorders may significantly increase the risk of developing IBD. Additionally, in IBD patients, Toll-like receptors (TLRs) produced by intestinal epithelial cells and dendritic cells treat intestinal bacterial antigens as pathogens, which causes a disruption of the immune response, resulting in the development of an inflammatory process. This may result in the occurrence of intestinal dysbiosis, which IBD patients are significantly vulnerable to. In this study, we reviewed scientific studies (in particular, systematic reviews with meta-analyses, being studies with the highest level of evidence) regarding the microflora of patients with IBD vs. the microflora in healthy people, and the use of various strains in IBD therapy.

## 1. Introduction

Inflammatory bowel diseases (IBDs) are chronic diseases of the gastrointestinal tract with a multifactorial and incompletely understood etiology. The peak incidence is observed during adolescence and in young adults, in whom, among other events, multiple interactions of the microbiota with the immune system lead to an inadequate immune response of the intestinal mucosa [1]. IBD more often affects Caucasian populations in industrialized and highly developed countries. Despite the heterogeneous incidence of IBD worldwide, the highest is found among residents of North America, Australia, and the northern part of Europe [1]. We can distinguish the two most common disease entities, namely Crohn’s disease (CD) and ulcerative colitis (UC). In addition, there is also one much rarer disease entity classified as inflammatory bowel disease and referred to as unspecified inflammatory bowel disease. In this subtype, the inflammatory features of the intestinal mucosa are more general and will not enable to classify as UC or CD [2]. Moreover, all symptoms typical to both disease entities mentioned above may alternate [1,2,3,4,5]. 

Ulcerative colitis occurs with periods of exacerbations and remissions. The inflammation is limited to the colon and rectum and only affects the intestinal mucosa. During long-term exacerbation, ulcers form in places with active inflammation. The intestinal wall thickens, and the physiologically occurring gastric tracts of the large intestine may disappear; however, granules do not form as in the case of CD. The severity of relapses and disease activity determines the therapeutic approach and prognosis, and regardless of age, all patients are treated depending on the course of the disease [4,5]. 

In turn, Crohn’s disease (CD) is a non-specific granulomatous inflammation of a segmental nature that may affect the entire digestive system, from the mouth to the anus. The lesions are solid, and inflammation begins in the mucosa, but as the inflammation progresses, it gradually affects the deeper layers of the intestinal wall and the mesentery. This causes their destruction, which, in the further course of the disease, leads to the formation of deep ulcers followed by strictures, abscesses, and fistulas. Similarly to UC, this disease progresses with periods of exacerbations and remissions [4,5].

The detailed characteristics of UC and CD are shown in Table 1. The classification of UC according to the extent of lesions and the number and severity of symptoms is shown in Supplementary Tables S1 and S2. In turn, the classification of CD stages is prefaced in Table S3. 

In this study, we reviewed scientific studies (in particular, systematic reviews with meta-analyses, being studies with the highest level of evidence) regarding the microflora of patients with IBD vs. the microflora in healthy people, and the use of various strains in IBD therapy. During this review, we have compared peer-reviewed scientific studies regarding the implementation of various probiotic strains in IBD therapy and their impact on microflora and health. We have focused on systematic reviews with meta-analysis since these have the highest levels of evidence.

## 2. Etiological Factors

**Figure 1:**
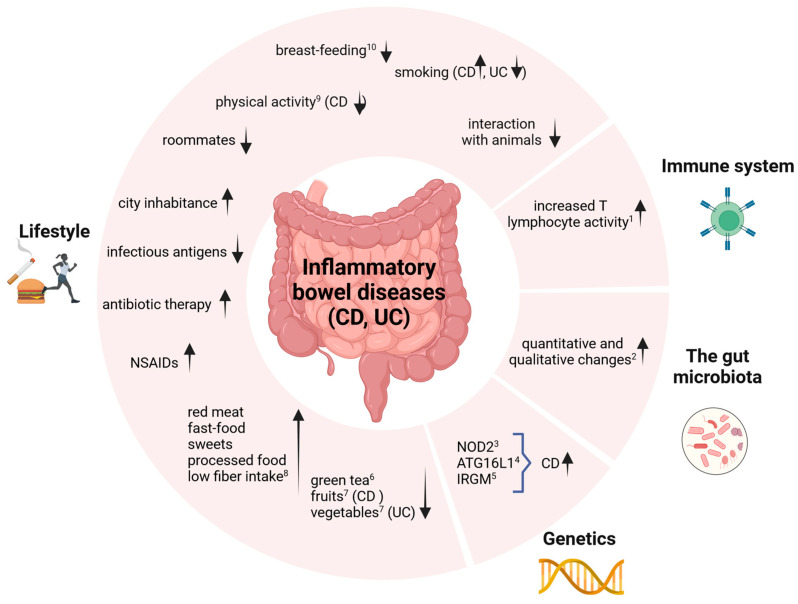
Factors influencing the development of inflammatory bowel disease (IBD). Created with BioRender.com. ↑ higher risk; ↓ lower risk; ATG16L1, autophagy-related-16-like-1; CD, Crohn’s disease; IRGM, immunity-related GTPase M; NOD2, nucleotide oligomerization binding domain 2; NSAIDs, non-steroidal anti-inflammatory drugs; UC, ulcerative colitis. (1) Th2 activity is increased in UC, and Th1 activity is increased in CD; both of these subpopulations produce interleukins, the excess of which causes an inappropriately strong humoral immune response, for which antibodies are responsible. As a result of the predominance of pro-inflammatory cytokines (tumor necrosis factor-alpha (TNFα), interleukin-6 (IL6), and interleukin-12 (IL12)) over anti-inflammatory cytokines, inflammation develops. (2) Increased ratios of strains from the Enterobacteriaceae and Bacteroides families in UC patients. (3) NOD2 (nucleotide oligomerization binding domain 2), a gene inherited in an autosomal, recessive manner. It is responsible for coding proteins involved in the non-specific immune response to bacterial antigens. (4) ATG16L1 (autophagy-related-16-like-1), similarly to NOD2, is a gene responsible for an abnormal immune response to intracellular bacteria. (5) IRGM (immunity-related GTPase M), similarly to NOD2, is a gene responsible for an abnormal immune response to intracellular bacteria. (6) Anti-inflammatory properties of green tea polyphenols were comparable to the effects of sulfasalazine on colitis. (7) Flavonoids contained in plants may play an important role in the proper functioning of the intestinal barrier, the disruption of which can be observed in IBD patients. (8) May result in a reduced amount of short-chain fatty acids produced by intestinal bacteria. (9) Normalizes the impaired autophagy process and reduces the concentration of pro-inflammatory cytokines. (10) The longer the breastfeeding period, the lower the risk of child inflammatory bowel disease; lack of breastfeeding was correlated with more frequent Clostridium difficile colonization and the occurrence of immunization diseases [6].

Inflammatory bowel disease has an incompletely understood pathogenesis. However, recent research suggests that it is a multifactorial process, and it is impossible to determine exactly why a given patient will develop inflammation [4]. The etiopathological factors include genetic, immunological, and lifestyle factors. The existing research also suggests that IBD is caused by an inappropriate interaction between the gut microbiota and the host’s immune system. Another important aspect is the dysfunction of the intestinal epithelium and its abnormal immune response to the changed microbiota [4,5,6]. The set of etiological factors known to date that may influence the development of the disease is shown in Figure 1. 

The presented factors play an important role in the modulation of the intestinal microbiota, leading to the conclusion that disturbances in the intestinal microflora may significantly increase the risk of developing IBD. The gut microflora is the main link between the body’s immune response and other risk factors [6]. 

## 3. Relationship of IBD with Gut Microflora and Immune System

The intestinal microflora has a significant impact on the appropriate development of innate and adaptive responses of the human immune system. The normalization of immune responses by gut bacteria is linked to Toll-like receptors (TLRs), which play a binding role between innate and acquired immunity [7,8,9]. 

The genesis of inflammatory bowel disease is closely related to the ability of the host’s immune system to distinguish between pathogenic bacteria and those that have no harmful effects on the body. Thus, it can be presumed that patients suffering from inflammatory bowel disease have partially lost the ability to distinguish between these two groups of bacteria [9]. The immune response is impaired so that TLR receptors, whose expression occurs in intestinal epithelial cells and dendritic cells, treat intestinal bacterial antigens as pathogens. TLR2 and TLR4 receptors play a significant role in maintaining homeostasis within the intestinal epithelium, indicating that their overexpression is associated with the formation and occurrence of active stages of inflammatory bowel disease [8]. 

## 4. Gut Microbiota in Patients with Inflammatory Bowel Disease

A significant proportion of IBD patients struggle with intestinal dysbiosis. This can be caused by a disruption in bowel movement frequency and an elimination diet, often a low-fiber diet. This increases the possibility that the metabolism of resident microorganisms in the gut may be disrupted, which in turn may have an impact on the pathogenesis of IBD [8,10]. The reduced number of probiotic bacteria in the intestine limits the amount of short-chain fatty acids they produce [10,11]. Acetic acid is the substrate for butyric acid, which is the primary source of energy for colonocytes, and its deficiency can significantly increase intestinal epithelial permeability. Interestingly, a study of the feces of people with IBD showed a reduced number of butyrate-producing *Firmicutes* and *Bacteroides* bacteria [11]. According to the researchers, short-chain fatty acids have a direct effect on inflammation reduction in the gastrointestinal tract. A careful analysis of the type and quantity of microorganisms in patients with IBD showed that they may contribute to the specific regulation of T lymphocytes [8,11,12]. Disturbed intestinal tightness has been directly linked to emerging intestinal conditions [10]. In addition, enzymes produced by microbiota isolated from the gut of IBD and obese patients simultaneously participate in the metabolism and membrane transport of nutrients [12]. 

A meta-analysis by Prosberg et al. in 2016 [13] showed that the microflora of patients with active disease compared to those in persistent remission differs significantly. Patients were shown to have a reduced abundance of *Clostridium coccoides* (MD = −0.49, 95% CI: −0.79 to −0.19, I^2^ = 0.0%, *p* = 0.925), *Clostridium leptum* (MD = −0.44, 95% CI: −0.74 to −0.14 I^2^ = 0.0%, *p* = 0.699), *Faecalibacterium prausnitzii* (MD = −0.81, 95% CI: −1.23 to −0.39; I^2^ = 62.1%, *p* = 0.007), and *Bifidobacterium* (MD = −0.37, 95% CI: −0.56 to −0.17; I^2^ = 0.0%, *p* = 0.572) [13]. 

On the other hand, a meta-analysis designed to test whether the abundance of bacteria of the genus *Bacteroides* differs in patients with inflammatory bowel disease compared to healthy subjects showed that there are significantly fewer of this type of bacteria in the intestines of patients diagnosed with IBD in both CU (MD = −0.68, 95% CI: −1.12, −0.35; I^2^ = 94%, *p* < 0.0001) and CD (MD = −1.62, 95% CI: −2.24, −1.01; I^2^ = NA, *p* < 0.00001) [14]. Importantly, the correct proportion of Bacteroides may favorably increase the levels of regulatory T cells and cytokines that protect against the development of colonic inflammation [15]. 

Another argument for the influence of intestinal dysbiosis on the occurrence and course of inflammatory bowel disease is that the use of antibiotics alters the abundance and ratio of bacteria in the intestinal lumen. Depending on the antibiotic administered, studies have shown this may have either contributed beneficially to or hindered disease remission [8,10,16]. The use of some antibiotics has been associated with dysbiosis, an increased risk of *C. difficile* infection, and reduced treatment efficacy [10]. Some studies have also found that intestinal reservoir mucositis, which is a common complication of UC in patients after ileal anastomosis, correlated with reduced amounts of *Lactobacillus* and *Bifidobacteria* inside the reservoir [16,17]. 

It is considered important that an adequate ratio and amount of non-pathogenic bacteria in the intestine increases the absorption of certain micronutrients. These include calcium and iron, adequate concentrations of which may have a positive effect on the course of inflammatory bowel diseases [16].

## 5. Importance of Particular Strains of Probiotic Microorganisms in the Treatment of Inflammatory Bowel Diseases

The World Health Organization (WHO) defines probiotics as live microorganisms that, when taken in adequate amounts, provide health benefits to the host. Their mechanisms of action and their effects vary and depend on the specific strain and dose. Probiotics can interact with the body in various ways. Some have a direct antimicrobial effect, producing substances such as bacteriocins or beta-defensins. Other microorganisms, on the other hand, act in a non-immunological way, competing with pathogens for nutrients, changing the pH of the intestines, and increasing the production of mucus in the intestinal lumen, which benefits tissue repair processes [18,19]. Metabolites produced by microorganisms such as short-chain fatty acids (SCFAs) nourish intestinal cells (colonocytes), strengthening tight junctions, which reduces the permeability of the intestinal mucosa. This is of great importance in IBD, which is characterized by an unsealed intestinal barrier [11,19]. The results of clinical trials on the use of probiotics in patients with IBD are inconclusive. Reports on the efficacy of probiotics in inducing or maintaining remission in IBD are conflicting, which may be due to the diversity of bacterial species or strains used as probiotics, as well as methodological differences between studies. However, this is an extremely important topic worth exploring due to the increasing incidence among young people seeking alternative methods to support drug treatment [1,11,19].

The specific mechanisms and effects of probiotics are strain- and dose-dependent. Some of them can produce substances that directly counteract other microorganisms. Others, on the other hand, can act in a non-immunological way to benefit the host by competing with pathogenic microorganisms for nutrients and space, altering the acid reaction of the intestine, enhancing mucus production, and supporting the repair processes of intestinal tissue [18,19]. This is why it is so important to know and properly match the strain of probiotic microorganisms to the patient’s needs and ailments [10,18,19]. 

To date, probiotics have been shown to have a moderate beneficial effect against UC and inflammation of the intestinal reservoir mucosa (pouchitis) [20]. 

A meta-analysis by Shen et al. [17] showed, based on twenty-three randomized trials with a control group, that appropriately administered probiotic therapy has a significant effect on the occurrence of remission in patients with UC (RR = 1.51; 95% CI (1.10; 2.06), *p* = 0.01; I^2^ = 65%, *p* = 0.004). In addition, an analysis of 16 remission maintenance studies showed the effectiveness of probiotic use in reducing the incidence of disease re-exacerbation (RR = 0.73; 95% CI (0.54; 0.99), *p* = 0.04; I^2^ = 59%, *p* = 0.001). A reduced number of symptom relapses was observed in the group of subjects who took the probiotic. An analysis of the group of UC patients who took probiotics showed significantly better effects in terms of maintaining and achieving remission compared to the control group taking a placebo (RR = 1.80; 95% CI (1.36; 2.39), *p* < 0.0001; I^2^ = 4%, *p* = 0.39) [17]. 

Crucially, after analyzing eligible studies without significant heterogeneity (I^2^ = 17%; *p* = 0.29), it was found that there were no adverse events with the use of probiotics. This is an undeniable advantage of using this type of therapy [17]. 

The results of a meta-analysis by Zhang et al. [21] showed that the use of probiotic preparations, prebiotic preparations, and also synbiotics supports the maintenance of remission in inflammatory bowel disease. However, the greatest difference was observed when patients used synbiotic preparations (RR = 1.39, 95% CI (1.05, 1.85), *p* < 0.05; I^2^ = 0%, *p* = 0.969). At the same time, the most effective type of therapy is the combination of pro-, pre-, or synbiotic products with drug therapy versus the use of drugs alone (RR = 1.14, 95% CI (1.02, 1.27), *p* < 0.05; I^2^ = 35.9%, *p* = 0.154). The study also showed that multi-strain formulations performed best (RR = 1.68, 95% CI (1.22, 2.29), *p* < 0.05; I^2^ = 55.7%, *p* = 0.035), and the reference dose to consume in alleviating IBD was 1010-1012 CFU/day (RR = 1.20, 95% CI (1.00, 1.44), *p* < 0.05; I^2^ = 64.8%, *p* = 0.002). An analysis of the collected studies showed that remission correlated significantly more often with the supplementation of pro-, pre-, or synbiotic products in patients with UC (RR = 1.16, 95% CI (1.02, 1.32), *p* < 0.05; I^2^ = 61.7%, *p* < 0.001). However, there was no significant relationship between the use of prebiotic, probiotic, or symbiotic products and the occurrence of relapse [21]. 

### 5.1. Saccharomyces Boulardii

These are yeast cultures that show an inhibitory effect on diarrhea, mainly those that occur after antibiotic therapy. Such an effect was shown in both children and adults, where the control group received no treatment, and the study groups received either a placebo or the probiotic *Saccharomyces boulardii* [22]. The risk of diarrhea decreased from 20.9% to 8.8% (RR: 0.36, 95% CI: 0.21–0.61, *p* = 0.0002; I^2^ = 46%, *p* = 0.013) in the probiotic-treated children group and from 17.4% to 8.2% (RR: 0.52, 95% CI: 0.36–0.73, *p* = 0.0002; I^2^ = 48%, *p* = 0.05) in the adult group. The meta-analysis also examined the effect of *S. boulardii* on the prevention of diarrhea caused by *C. difficile* infection. They showed a reduced risk of diarrhea among children using *S. boulardii* compared to a group of children taking a placebo (RR = 0.25, 95% Cl 0.08–0.73, *p* = 0.01; I^2^ = 0%, *p* = 0.44). However, a significant relationship was not shown among adults [22]. 

The results of a study by Plein et al. indicate a significant reduction in bowel movements in Crohn’s disease patients after 7 weeks of taking 750 mg of *S. boulardii* (1.5 × 10^10^/d) (mean = 3.3 ± 1.2 stools/d, *p* < 0.05) compared to the placebo group (mean= 4.6 ± 1.9 stools/d) [23]. 

*S. boulardii* may be a suitable option for people struggling with persistent diarrhea, but it is worth bearing in mind that *S. boulardii* preparations should not be used in people with serious comorbidities, those with central venous catheters, and also those in the intensive care unit, as there is a risk of fungemia [24]. 

### 5.2. Lactic Acid Bacteria

Lactic acid bacteria (LAB) and *Bifidobacterium* can produce lactic acid, which is the end product of carbohydrate (mainly lactose) fermentation. They can eliminate emerging abnormalities in the composition of the intestinal microbiota caused by the use of antibiotic therapy or existing gastrointestinal dysfunctions such as IBD. They also show increased tolerance to an acidic environment. During fermentation, LAB can produce numerous peptides that have beneficial effects on the body. These include antioxidant peptides, antimutagenic peptides, and also angiotensin-converting enzyme I (ACE-I), which is involved in blood pressure regulation [8,25,26]. 

A randomized, double-blind study in which researchers administered *Lactobacillus rhamnosus GG* (LGG) or a placebo immediately after the removal of the diseased portion of the intestine in people with CD showed no significant differences in the maintenance of endoscopic and clinical remission in the two groups during 12 months [8]. A similar study was conducted in a group of children with CD. The children were divided into a group receiving LGG and a placebo group, with both groups concurrently taking drugs such as aminosalicylates, azathioprine, corticosteroids, and low-dose 6-mercaptopurine. The study lasted 2 years, and while the group of children taking the probiotic achieved remission faster than the placebo group, relapse was also noted in a higher percentage of this group. However, the differences between the sets were not statistically significant. The researchers argue that this is a result of the increased resistance to colonization of the LGG strain in CD patients. It is possible that with a different dose or a different strain of *Lactobacillus*, the results could be different [8].

### 5.3. Escherichia coli Strain Nissle 1917

*Escherichia coli* strain Nissle 1917 (EcN) is a non-pathogenic Gram-negative bacterium that has found its use in alleviating the symptoms of gastrointestinal diseases such as colonic diverticulosis and inflammatory bowel disease, particularly UC. It was isolated in 1917 from the bacterial flora of one soldier who, unlike others, had no symptoms indicative of infection with the then-prevalent Shigella in the Balkans. A probiotic drug was quickly developed from the isolated strain, whose availability has so far been limited to Germany and Italy [27,28]. EcN, under normal conditions, colonizes the intestine in just a few days and can reside there for up to several months after administration. EcN can affect the entire human system, with a focus on anti-inflammatory effects on the gut [27]. Due to its antibacterial properties, it can inhibit the colonization of EHEC (a pathogenic form of *E. coli*) and, through biofilm formation, inhibit the synthesis of the Shiga toxin responsible for bacterial infections of the gastrointestinal tract. *E. coli Nissle 1917* has a filament that acts as a drive, giving EcN an advantage in competing with pathogens for space in the host body. It also stimulates intestinal epithelial cells to produce defensin, whose action focuses on inhibiting the adhesion of pathogenic forms of *E. coli* to intestinal cells [27,29,30]. This is important because pathogenic *E. coli* bacteria are triggers of the immune response in patients with IBD. Among the beneficial properties of EcN, its ability to seal the intestinal wall is also important. The strengthening of connections between intestinal epithelial cells is due to the increased expression of mRNA by *E. coli Nissle 1917* bacteria. Its anti-inflammatory effect is based on reducing the concentration of pro-inflammatory cytokines and increasing the concentration of anti-inflammatory cytokines through a corresponding effect on the immune system [27,28]. 

In a study with mice, acute colitis was induced using dextran sulfate sodium (DSS), and chronic colitis was induced by transferrin CD4+CD62L T cells. In immunocompromised (SCID) mice, EcN was shown to be effective in alleviating the symptoms of colitis, but only in a chronic inflammation model [28]. EcN activity was associated with a reduction in the amount of pro-inflammatory cytokines secreted. There are also studies suggesting a symptom-relieving effect and reduced weight loss in mice treated with DSS and EcN treatment. At the same time, studies were conducted to see if the Nissle 1917 strain could be as effective as mesalazine for patients with ulcerative colitis. Three independent double-blind, double-sham studies were conducted on adult patients with UC [29,30,31]. The subjects were divided into groups taking mesalazine and the probiotic *E. coli Nissle 1917*. All three studies showed an equivalence in the effects of the two drugs with the absence of adverse events in both groups. There was little difference in the results, and tolerance to both preparations was good to very good. Patients’ condition was monitored very closely with indices of disease activity on endoscopic and histological imaging. Patients’ subjective opinion of their quality of life was also assessed, which did not differ between groups after the study. The study confirmed that the *E. coli Nissle 1917* strain can provide beneficial effects in the treatment of UC [29,30,31]. The undeniable disadvantage of this strain is its low availability, which is limited only to certain European Union countries [27]. 

A meta-analysis [32] conducted on the efficacy of UC cast treatment with *E. coli Nissle 1917* showed that EcN-induced remission was achieved in 61.6% of cases, with 69.5% in the control group taking mesalazine (MD = 7.9%, OR = 0.92; 95% CI 0.15–9.66, *p* = 0.93). Due to the high heterogeneity (I^2^ = 86%; *p* = 0.001), a random effect model was used. Re-exacerbation occurred in 36.1% of the control group (mesalazine) and 36.8% in the group taking the EcN formulation (MD = 0.8%, OR = 1.07, 95% CI 0.70–1.64, *p* = 0.74; I^2^ = 0%, *p* = 0.82). Conclusions drawn following the above meta-analysis, if they should be supported by further studies, indicate that the use of an *E. coli Nissle 1917* preparation may be similarly effective in inducing and maintaining remission in UC to that of the standard mesalazine therapy [32].

### 5.4. A Mixture of Bacterial Strains from the Genus Bifidobacterium, Lactobacillus, and Streptococcus

Several studies of probiotics consisting of a mixture of bacterial strains from the genus *Lactobacillus, Bifidobacterium*, and *Streptococcus* have shown that their administration to patients with IBD has a beneficial effect on achieving remission and alleviating disease symptoms [20,33,34]. 

Some have also suggested that they may have anti-cancer effects, which may carry additional benefits due to the increased risk of gastrointestinal carcinogenesis in patients with IBD [34,35]. 

A 2018 study [34] by Chinese researchers investigated if treatment with the probiotic VSL#3 would have a positive effect in mice with azoxymethane/dextran sodium sulfate (AOM/DSS)-induced recurrent colitis [35]. AOM/DSS exhibit carcinogenic effects, and tumors caused by their administration can develop in up to 10 weeks. The VSL#3 probiotic used in the study consisted of strains such as *Lactobacillus casei*, *Lactobacillus plantarum*, *Lactobacillus acidophilus*, *Lactobacillus delbrueckii* subsp. *bulgaricus*, *Bifidobacterium longum*, *Bifidobacterium breve*, *Bifidobacterium infantis*, and *Streptococcus salivarius* (*thermophilus*). The mice were divided into five groups where the first was a control group (without inflammation induction), the second received no treatment, the third received 5-aminosalicylic acid (mesalazine, 5-ASA), the fourth VSL#3, and the fifth both 5-ASA and VSL#3. All mice treated with the inflammation inducer showed symptoms such as weight loss, nausea, blood in the stool, and a change in its consistency. The aforementioned symptoms were not observed in the control group. All mice not in the control group developed a malignant tumor within the colon. It was noted that in the group that received 5-ASA and VSL#3, both the tumor growth rate and the number of tumor cells were reduced. Pro-inflammatory markers such as TNFα and IL-6 were also reduced, and the mice were also tested for the composition of their intestinal microflora. While there were no significant differences before the study, after administration of AOM/DSS, there was a reduced amount of *Lactobacillus* with an increase in *Oscillibacter* and *Lachnoclostridium* [34]. It is noteworthy that the *Lactobacillus* family bacteria may have beneficial effects on intestinal inflammation. *Lactobacillus bulgaricus* and *Lactobacillus rhamnosus* have been shown to reduce symptoms and maintain remission in UC. In contrast, *Oscillibacter* and *Lachnoclostridium* are recently discovered pathogens associated with gastrointestinal diseases [34]. 

After completion of the study, it was shown that the group receiving VSL#3 and 5-ASA had significantly elevated concentrations of *Bacillus* and *Lactococcus*, while *Oscillibacter* and *Lachnoclostrium* were reduced in the intestinal microbiota compared to the group without the introduced treatment. Most importantly, all differences regarding microbiota composition in the mice studied were statistically significant [34]. 

Throughout the study, it was proven that VSL#3 administration significantly reduces the rate of proliferation and the risk of tumorigenesis. Thus, the researchers conclude that VSL#3 administration may prevent carcinogenesis in patients with ulcerative colitis by reducing pro-inflammatory factors [34]. 

In a 2020 Italian study, researchers continued their work on the effects of different strain combinations on the course and maintenance of inflammatory bowel disease remission [20]. 

Mice were subjected to disease-inducing 2,4,6-trinitrobenzene sulfonic acid (TNBS) or dextran sodium sulfate (DSS) to induce colitis. Assigned to two groups based on the inflammation induction model, the mice were divided into subgroups according to the probiotic administered. Separate subgroups were those receiving no treatment, those receiving the probiotic Vivomixx, a single strain of *Bacillus subtilis*, Vivomixx along with *B. subtilis*, and a subgroup receiving an innovative mixture of five strains [20]. 

The bacteria found in the probiotic of the five strains included the following (percentage found): *Streptococcus thermophilus* (30%), *Lactobacillus casei* (30%), *Bifidobacterium breve* (15%), *Bifidobacterium animalis subsp. Lactis* (15%), and *Bacillus subtilis* (10%). Vivomixx was chosen to see if, as the next generation of the VSL#3 probiotic, it could work effectively in areas where VSL#3 had not shown effectiveness. The strains included in Vivomixx were as follows: *Streptococcus thermophilus*, *Lactobacillus plantarum*, *Bifidobacterium breve*, *Lactobacillus paracasei*, *Lactobacillus delbrueckii* subsp. *Bulgaricus*, *Lactobacillus acidophilus*, *Bifidobacterium longum*, and *Bifidobacterium infantis* [20]. 

A group of mice receiving TNBS, the effect of which induces symptoms similar to chL-C, indicated a preventive effect of Vivomixx. However, the effect achieved by administering the five-strain probiotic was more effective. There was an inhibition of weight loss by about 15% and a 45% alleviation of inflammatory activity as measured by the Crohn’s disease activity index (CDAI ) index compared to TNBS-treated mice without treatment. The micro- and macroscopic picture of inflammation was also significantly improved in this group. The combination of VSL#3 with *B. subtilis* enhanced the effect of Vivomixx but not enough to surpass that of the five-strain probiotic. In contrast, the administration of *B. subtilis* alone did not significantly affect the condition of the mice. The above conclusions were supported by examining the expression of biomarkers showing pro-inflammatory and anti-inflammatory effects in the colon and mesenteric lymph nodes. An analysis of the concentration of pro- and anti-inflammatory biomarkers showed an increased amount of pro-inflammatory mediators in mice receiving TNBS alone by a factor of up to 30 times. At the same time, a significant advantage of the five-strain formulation over Vivomixx was noted concerning its ability to lower interleukin-6 (IL-6) levels. Nevertheless, both formulations reduced the concentration of pro-inflammatory cytokines while increasing the expression of anti-inflammatory mediators [20]. 

In order to test the efficacy of Vivomixx and the five-strain preparation against chronic colitis, mice were administered DSS along with drinking water and then administered the probiotic preparation according to subgroup. Both Vivomixx and the five-strain formulation were proven to reduce colitis symptoms to the same extent as assessed by body weight measurements, CDAI, and pro-inflammatory biomarker levels [20]. However, only the probiotics of the five strains showed the ability to normalize the total white blood cell count and lymphocyte counts, whose concentrations increased DSS. Based on histopathological evaluation of colon sections, it was shown that both Vivomixx and the preparation of the five strains inhibited the pathological changes occurring in the intestine under the influence of DSS. The pathological changes observed in macroscopic and microscopic examination of the intestine were attenuated, which showed that administration using the five strains was more effective than Vivomixx. An analysis of biomarkers among mice exposed to DSS showed a similar relationship to that of the TNBS group. Increased expression of inflammatory mediators was attenuated with both probiotic preparations. However, better results, confirming the anti-inflammatory effect, were obtained after using the probiotic of five strains. After analyzing the composition of the intestinal microbiota of mice after administration of DSS and after completion of probiotic therapy, it was found that the composition of the microbiota worsened significantly in each individual after receiving DSS, and its modification after probiotic administration depended on the preparation [20]. 

A meta-analysis conducted by Mardini et al. [36] to analyze the efficacy of the probiotic VSL#3 as adjunctive therapy for the induction of remission in patients with mild to moderate exacerbation of UC showed that a decrease in ulcerative colitis disease activity index (UCDAI) of >50% was demonstrated by 44.6% of patients receiving VSL#3 versus 25.1% of patients in the placebo group ( OR = 2.793; 95% CI, 1.375–5.676; *p* = 0.008). In contrast, disease remission was achieved by 43.8% of patients taking VSL#3 versus 24.8% of the placebo group (OR = 2.4; 95% CI, 1.48–3.88; *p* = 0.007). Both groups were concurrently taking the standard 5-ASA and/or immunomodulators used in IBD therapy, and the daily dose of VSL#3 was 3.6 × 1012 CFU/d. The heterogeneity test showed low heterogeneity between the analyzed studies (I^2^ = 38%, *p* = 0.22 for >50% decrease in UCDAI and I^2^ = 29%, *p* = 0.24 for remissions achieved) [36]. 

The above results were confirmed by a meta-analysis from 2020 by Dang et al. [37] on the efficacy of using VSL#3 in combination with drug therapy in patients with mild to moderate UC. The use of VSL#3 significantly provided an improved clinical response in patients with UC (OR = 3.09, 95% CI = 1.53–6.25, *p* < 0.001, I^2^ = 46%, *p* = 0.16) without any significant side effects (OR = 0.90, 95% CI 0.33–2.49, *p* = 0.84) [37]. 

## 6. Use of Prebiotics in IBD

Prebiotics are nutrients that are not digested in the intestinal lumen. However, they have an extremely important function. They nourish specific bacteria populating the intestine by potentiating their growth and metabolic processes. This results in increased production of SCFAs in the intestine and also the growth of beneficial microorganisms that are so important in the fight against digestive diseases [38].

The main groups of prebiotics include fructooligosaccharides (FOSs), galactooligosaccharides (GOSs), and inulin and lactulose [38]. Despite many studies on the beneficial effects of prebiotics in healthy individuals, there are still not enough results relating to the use of such products by IBD patients. In a study conducted by Benjamin et al. [39], patients with exacerbation of CD noted clinical deterioration after 15g of FOS for 4 weeks. In contrast, a study by Hafer et al. showed an adverse effect of 10g lactulose supply to patients with an active form of IBD. Supplementation lasted for 4 months. No favorable changes in endoscopic imaging or clinical disease activity were observed after the supplementation period ended [40]. In a recent 2024 systematic review, researchers showed that fructooligosaccharide (FOS) kestose was effective in inducing UC remission (RR: 2.75, 95% CI 1.05–7.20; n = 40). In contrast, the supply of oligofructose-enriched inulin had no beneficial effect. Similar findings were observed for CD, with FOS being significantly less effective than for UC. Patients frequently reported experiencing bloating and abdominal overflow [41].

An interesting prebiotic product to discuss is arabinogalactan, which may prove to be a suitable choice for patients with IBD.

Arabinogalactan (AG) is a highly safe, soluble dietary fiber found abundantly in coniferous trees, e.g., *Mangifer aindica* L., Astralagus gummifer, and larch wood. AG has anti-inflammatory, antioxidant, immune-boosting, and anti-cancer properties and is utilized by human gut bacteria such as Bifidobacterium and Bacteroides species. AGs enrich the abundance of Bacteroides cellulosilyticus in single-strain co-cultures, in vitro fermentations, and in vivo mouse models. In the study by Gao et al. [42], AG supplementation ameliorated colitis and clinical symptoms in mice. Another study found that arabinogalactan proteins (AGPs) in wheat flour increased bifidobacteria and short-chain fatty acid concentrations in a batch culture of human feces. Improving the number of Bacteroides cellulosilyticus and Bifidobacteria is a feasible method of treating inflammatory bowel disease (IBD) [42].

In turn, Zheng et al. [43] showed that AG in Caco-2 cells inhibited oxidative stress and inflammation by activating AMPK/SIRT1 and inhibiting the NF-B signaling pathway, thereby promoting tight junction (TJ) protein expression and alleviating intestinal epithelial barrier (IEB) damage by LPS. The integrity of the TJ is destroyed under the influence of harmful substances such as reactive oxygen species (ROS), cell byproducts that initiate oxidative stress and cell damage), which may result in excessive activation of the inflammatory response, therefore maintaining the integrity of the IEB may be an effective method to alleviate IBD. This suggests that AG may be a functional food additive to alleviate IBD [43].

## 7. Discussion

Recent studies indicate that the state of the gut microbiota plays a significant role in maintaining homeostasis in the host body [40]. The same relationship has been demonstrated in inflammatory bowel diseases. The composition and quality of the resident gut microbiota are important in achieving and maintaining remission in IBD [6]. Importantly, the validity of introducing a properly selected probiotic therapy into the treatment of IBD patients was proven in the study conducted by Prosberg et al., in which the composition of the intestinal microflora in IBD patients was altered compared to healthy individuals [13]. The discovery of these differences made it possible to put forward a thesis regarding changes in the microbiota as one of the main pathogenetic factors in these disease entities. Meta-analyses conducted over the past few years have shown that the use of right-sizing probiotic therapy in terms of dose, number of strains, and also auxiliary components such as prebiotics had a statistically significant more favorable effect than a placebo in inducing and maintaining disease remission [17,21,22,23,34,36]. These studies were more likely to focus their attention on the validity of this type of support in UC patients. The effect of probiotic therapy on the course of the disease was limited in patients with CD. 

Depending on the strain used, the therapeutic effect was different, as shown in meta-analyses conducted by different researchers in the last 10 years. It was shown that *S. boulardii* significantly reduces the incidence of post-antibiotic diarrhea and the risk of *C. difficile* infection, which is common and difficult to treat for IBD patients [22,23,24]. The use of *E. coli* Nissle 1917 in UC patients shows similar effects to the standard mesalazine treatment [29,30,31]. The VSL#3 multi-strain probiotic, which includes strains such as Lactobacillus casei, Lactobacillus plantarum, Lactobacillus acidophilus, Lactobacillus delbrueckii subsp. bulgaricus, Bifidobacterium longum, Bifidobacterium breve, Bifidobacterium infantis, and Streptococcus salivarius (thermophilus) also achieved satisfactory results in inducing remission when used concurrently with primary pharmacotherapy [36]. What distinguished the probiotic from other types of treatment were the negligible side effects limited to gastrointestinal disorders [17,24]. This allows us to conclude that it is a relatively safe type of therapy to use with caution regarding dosage and duration of supplementation. Our analysis of the available literature helped us to identify the parameters worth paying attention to when selecting a preparation for a patient with active IBD. It is reasonable to choose multi-strain preparations in the form of a synbiotic, which is a combination of probiotic bacteria with a well-matched substance with prebiotic activity [21]. In the conducted literature review, these preparations showed the greatest effectiveness. The duration of therapy was not statistically significant for a stronger or longer-lasting effect; however, most studies were conducted for 4 to 12 weeks of intervention. It is worth noting that most of the studies were conducted on animal models, which are significantly different from humans. Given the increasingly high incidence rate of IBD among young people worldwide and the optimistic results in studies conducted to date, further analysis and research conducted on the human population to select the most efficient strains for IBD are warranted.

## 8. Conclusions

The microbiota plays an important role in the etiology and course of inflammatory bowel diseases. The use of products containing probiotic strains significantly influenced patients’ well-being and disease activity index, and the best results were noticed when basic pharmacotherapy and probiotic or symbiotic products were used simultaneously. Probiotic therapy played a much greater role among patients with UC than CD. There are still not enough studies conducted on the human population that unequivocally confirm the appropriateness of probiotic supplementation in IBD.

## Figures and Tables

**Table 1:** Characteristics of inflammatory bowel diseases (IBDs) [3,4,5].

	Ulcerative Colitis (UC)	Crohn’s Disease (CD)
Gastrointestinal symptoms	Chronic diarrhea with admixture of fresh blood in almost 90% of patients; severe bleeding from the lower part of the gastrointestinal tract; sudden and intense pushing on the stool; expulsion of mucopurulent contents; severe abdominal pain of a spasmodic nature, located mostly in the left side of the iliac fossa, usually subsiding after defecation; in patients with the proctitis form, symptoms are usually limited to violent pushing on the stool with the presence of fresh blood; constipation instead of diarrhea may also occur.	Chronic, watery diarrhea, often with admixture of mucus and blood; often with occult blood from the bleeding small intestine; bowel movements also at night; with the constrictive form, the symptom is constipation with symptoms of incomplete obstruction; crampy abdominal pain located on the right side of the iliac fossa and near the umbilicus; flatulence; pain in the lower abdomen often occurs with a feeling of pushing on the stool; perianal lesions (including anal fissures and fistulas) that are asymptomatic or with burning pain at the anus aggravated during bowel movements and sitting; active fistulas discharge purulent contents; symptoms in the upper gastrointestinal region usually occur along with other symptoms (rarely the only complaints the patient has); lesions of the stomach and duodenum, nausea, vomiting, and pain in the epigastrium; pathologically involved esophagus—dysphagia and odynophagia (swallowing disorders, pain when swallowing); in the oral cavity, ulcers, as well as aphthous ulcers.
Other symptoms	With more severe episodes: fever, tachycardia, weight loss, nausea, and vomiting; osteoarticular system: arthritis, osteopenia, and osteoporosis; skin lesions (erythema nodosum, gangrenous dermatitis); pathological changes in the liver and biliary tract, such as hepatic steatosis and primary sclerosing cholangitis; venous thromboembolism.	Usually lasting chronically (more than 6 weeks): weakness, weight loss, lack of appetite, subfebrile states and fever, night sweats; extraintestinal symptoms: cholelithiasis—in up to 30% of patients with ileus, urolithiasis, clubbed fingers—occurs in 40–60% of patients with severe flares, pain in axial and peripheral joints and peripheral joints, erythema nodosum, pyoderma gangrenosum; in 3–5%, visual problems such as conjunctivitis, or iritis.
Long-term effects of the disease	Chronic inflammation of the colon strongly associated with an increased risk of colorectal cancer in the future, especially if the period of active stage of the disease is more than 10 years, which promotes dysplasia within the intestinal epithelium; the risk of malignancy increases, with concomitant primary sclerosing cholangitis [6]; pancolectomy is necessary in about 20% of patients (10 to 15% develop intestinal reservoir inflammation (pouchitis)).	Increased risk of future colorectal cancer in some cases; inflammatory subtype may develop into a fistulizing or narrowing form; most patients are forced to undergo surgery, which can lead to short bowel syndrome (nutritional deficiencies due to a smaller surface area for nutrient absorption); comorbidities and extraintestinal symptoms due to exacerbation or side effects of medications.

## Supplementary Materials

The following supporting information can be downloaded at https://www.mdpi.com/article/10.3390/nu16132092/s1, Table S1: Total Mayo score (TMS), disease activity index (DAI) for ulcerative colitis; Table S2: Classification of UC depending on the extent of the disease—Montreal classification. Table S3: Montreal Classification of Crohn’s disease. References [44,45] are cited in the Supplementary Materials.
