# Supplementary material for: Importance of Gut Microbiota in Patients with Inflammatory Bowel Disease
Source: Nutrients. 2024 Jun 30;16(13):2092. doi: 10.3390/nu16132092 (PMC11242987; doi:10.3390/nu16132092)
Supplement: Supplementary file 1 [file nutrients-16-02092-s001.zip › nutrients-3009708-supplementary.pdf]

Table S1. Total Mayo Score – TMS, Disease Activity Index (DAI) for Ulcerative Colitis [3,5,45].

Points	0	1	2	3
Stool frequency	Normal	1-2 stools/day more than normal	3-4 stools/day more than normal	>4 stools/day more than normal
Rectal bleeding	None	Visible blood with stool less than half the time	Visible blood with stool half of the time or more	Passing blood alone
Mucosal appearance at endoscopy	Normal or inactive disease	Mild disease (erythema, decreased vascular pattern, mild friability)	Moderate disease (marked erythema, absent vascular pattern, friability, erosions)	Severe disease (spontaneous bleeding, ulceration)
Physician rating of disease activity[1]	Normal	Mild	Moderate	Severe

[1] subjective assessment - mainly takes into account the presence of abdominal pain, the patient's general well-being, and the results of the physical examination. Interpretation: 0-2 points – remission (provided that none of the criteria was rated at 2 points); 3 - 5 points - mildly active disease; 6 - 10 points - moderately active disease; 11 - 12 points - severely active disease.

Table S2. Classification of UC depending on the extent of the disease - Montreal classification [5,45].

Type of Disease	Extent	Characteristic
E1	Proctitis	involvement limited to the rectum (rectosigmoid junction)
E2	Left-sided ulcerative colitis	involvement limited to the portion of colorectum distal to splenic flexure
E3	Extensive ulcerative colitis	involvement extends proximal to splenic flexure

Table S3. Montreal Classification of Crohn's disease [4, 44].

	Montreal Classification
Age of diagnosis – A	A1 <17 years<br>A2 17-40 years<br>A3 >40 years
Location – L	L1 – terminal ileum<br>L2 - colon<br>L3 – ileocolon<br>L4 – upper gastrointestinal *
Behaviour – B	B1 – non-stricturing, non-penetrating<br>B2 – stricturing<br>B3 – penetrating<br>p – perianal **

* L4 is a modifier that can be added to L1,2 and L3 when concomitant upper gastrointestinal disease is present
** P is a modifier that can be added to B1, 2 and 3 when concomitant perianal disease is present
